# Evaluation of a Frequency-Lowering Algorithm for Adults With High-Frequency Hearing Loss

**DOI:** 10.1177/2331216517734455

**Published:** 2017-10-13

**Authors:** Marina Salorio-Corbetto, Thomas Baer, Brian C. J. Moore

**Affiliations:** 1 Department of Experimental Psychology, University of Cambridge, UK

**Keywords:** frequency lowering, frequency transposition, dead regions, hearing aids

## Abstract

The objective was to determine the effects of a frequency-lowering algorithm (frequency composition, Fcomp) on consonant identification, word-final /s, z/ detection, the intelligibility of sentences in noise, and subjective benefit, for people with high-frequency hearing loss, including people with dead regions (DRs) in the cochlea. A single-blind randomized crossover design was used. Performance with Bernafon Acriva 9 hearing aids was compared with Fcomp off and Fcomp on. Participants wore the hearing aids in each condition in a counterbalanced order. Data were collected after at least 8 weeks of experience with a condition. Outcome measures were audibility, scores from the speech perception tests, and scores from a questionnaire comparing self-perceived hearing ability with Fcomp off and Fcomp on. Ten adults with mild to severe high-frequency hearing loss (seven with extensive DRs, one with patchy or restricted DRs, and two with no DR) were tested. Fcomp improved the audibility of high-frequency sounds for 6 out of 10 participants. There was no overall effect of Fcomp on consonant identification, but the pattern of consonant confusions varied across conditions and participants. For word-final /s, z/ detection, performance was significantly better with Fcomp on than with Fcomp off. Questionnaire scores showed no differences between conditions. In summary, Fcomp improved word-final /s, z/ detection. No benefit was found for the other measures.

## Introduction

### The Importance of Amplification at High Frequencies

It is well known that speech sounds characterized by high-frequency components carry a significant proportion of speech information (American National Standards Institute, 1997). Relatively strong high-frequency components occur for consonant groups such as the stops (/p, t, k, b, d, g/; Halle, Hughes, & Radley, 1957; Stevens & Blumstein, 1978), the fricatives (/f, ɵ, s, ʃ, h, v, z/; Hughes & Halle, 1956; Jongman, Wayland, & Wong, 2000), and the affricates (/dʒ, tʃ/; Stevens, 1993). Stops and fricatives, together with the nasal consonants /m, n/, account for 80% of all the consonantal distinctions among words for English (Pickett, 1999). For people with hearing loss, the intelligibility of speech in noise is correlated with the average hearing loss at 2 and 4 kHz (Smoorenburg, 1992) or 2, 3, 4, and 6 kHz (Amos & Humes, 2007). For a review, see Moore (2016). In addition, the audibility of high-frequency components is important for sound quality (Brennan et al., 2014; Moore, Füllgrabe, & Stone, 2011; Plyler & Fleck, 2006; Ricketts, Dittberner, & Johnson, 2008), detection of word-final /s, z/ (Füllgrabe, Baer, Stone, & Moore, 2010), and identification of speech in noise (Moore, 2016; Plyler & Fleck, 2006). A bandwidth of 5 kHz or more is required to discriminate /s/ from other high-frequency consonants in the speech of female and child talkers (Stelmachowicz, Pittman, Hoover, & Lewis, 2001). Access to high-frequency speech components also has long-term effects that can affect development. For children, adequate high-frequency amplification is important for the development of grammatical morphemes in speech (Koehlinger, Van Horne, Oleson, McCreery, & Moeller, 2015; McGuckian & Henry, 2007; Moeller et al., 2007).

Electroacoustic limitations of the receivers or the microphones of hearing aids and the shape of the long-term average spectrum of speech, which falls progressively with increasing frequency above 0.5 kHz (Moore, Stone, Füllgrabe, Glasberg, & Puria, 2008), often make it difficult to restore the audibility of high frequencies. For people with severe high-frequency hearing loss, it may be difficult to achieve the required gains and output levels without introducing distortion or encountering problems with acoustic feedback. Even when audibility is achieved, it does not always improve sound quality (Ricketts et al., 2008) or speech intelligibility (Baer, Moore, & Kluk, 2002; Hogan & Turner, 1998; Vickers, Moore, & Baer, 2001). Gain at high frequencies has sometimes been found to lead to decreased speech intelligibility (Ching, Dillon, & Byrne, 1998; Hogan & Turner, 1998; Rankovic, 1991). Hearing-impaired listeners often fail to obtain benefit from the provision of amplification at frequencies between 3.2 and 6.4 kHz, unlike normal-hearing control listeners (Amos & Humes, 2007), and this cannot be completely accounted for by the high presentation levels used, which decrease intelligibility even for normal-hearing listeners (Hogan & Turner, 1998). Most likely, the impaired ability to use audible information is related to a reduced ability to discriminate sounds, in some cases caused by the presence of regions of the cochlea with damaged or completely nonfunctioning inner hair cells, synapses, or neurons (Hogan & Turner, 1998; Ricketts et al., 2008). These are termed “dead regions” (DRs) in the cochlea (Moore, 2001, 2004). DRs most commonly affect the basal part of the cochlea (Cox, Alexander, Johnson, & Rivera, 2011; Vinay & Moore, 2007) and they are usually associated with hearing thresholds greater than 70 dB HL (Aazh & Moore, 2007; Vinay & Moore, 2007) and sloping hearing loss (Markessis, Kapadia, Munro, & Moore, 2006; Moore, 2004; Preminger, Carpenter, & Ziegler, 2005). When DRs are extensive—that is, they are present at three or more consecutive audiometric frequencies (Pepler, Munro, Lewis, & Kluk, 2014)—amplification of frequency components falling well within the DRs does not seem to be beneficial (Baer et al., 2002; Malicka, Munro, Baer, Baker, & Moore, 2013; Moore, 2002; Vickers et al., 2001).

### Conveying High-Frequency Information With Frequency-Lowering Hearing Aids

Frequency-lowering (FL) hearing aids lower the frequencies of components within a “source band” (SB) to place them in a “destination band” (DB) where the listener has better hearing. This type of processing may improve the ability to extract information about the components in the SB. A variety of methods can be used to achieve FL (Alexander, 2013; Braida et al., 1979; Simpson, 2009), including frequency compression (FC) and frequency transposition (FT).

**Figure 1.**
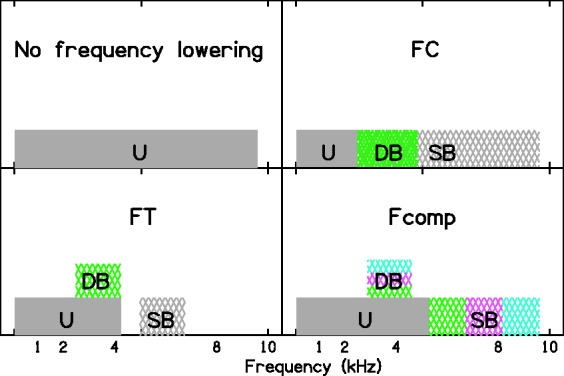
Schematic illustration of three forms of frequency lowering: frequency compression (FC, top right), frequency transposition (FT, bottom left), and frequency composition (Fcomp, bottom right). U = unprocessed (top left), SB = source band, DB = destination band. For FC, the SB (gray cross hatching) is wider than the DB (green cross hatching), and these bands have the same low-frequency edge. For FT, the SB (gray cross hatching) and the DB (green cross hatching) have the same width. For Fcomp, the SB is divided into three subbands, shown in different colors, and all subbands are transposed to the same DB. For FT and Fcomp, the frequency-lowered components are added to the unprocessed components.

With FC, the amount by which the frequency is lowered increases with increasing frequency within the SB, so that the width of the DB is less than the width of the SB; see the top-right panel of Figure 1. In FC systems, frequency components below a certain starting frequency, SF, are usually left unchanged, and the SB and DB are placed just above SF. Evaluations of FC systems have given mixed results. Improvements have been reported for sound detection (Glista et al., 2009; Wolfe et al., 2010, 2015), identification of closed-set consonants in quiet (Ellis & Munro, 2015b; Glista et al., 2009; Hopkins, Khanom, Dickinson, & Munro, 2014; Picou, Marcrum, & Ricketts, 2015; Simpson, Hersbach, & McDermott, 2005; Wolfe et al., 2010, 2011) and in noise (Ellis & Munro, 2015b), the intelligibility of sentences in noise (Bohnert, Nyffeler, & Keilmann, 2010; Ellis & Munro, 2015b; Wolfe et al., 2011), and word-final /s, z/ detection (Glista et al., 2009; Wolfe et al., 2010, 2011). However, some other studies indicated no benefits for some of the same outcome measures: sound detection (John et al., 2014), identification of closed-set consonants in quiet (Hillock-Dunn, Buss, Duncan, Roush, & Leibold, 2014; John et al., 2014; Kokx-Ryan et al., 2015; Simpson, Hersbach, & McDermott, 2006; Wolfe et al., 2015), identification of open-set consonants in quiet (Picou et al., 2015) and spondees in noise (Hillock-Dunn et al., 2014), word-final /s, z/ detection (John et al., 2014; Wolfe et al., 2015), and the intelligibility of sentences in noise (Hopkins et al., 2014; John et al., 2014; Kokx-Ryan et al., 2015; Picou et al., 2015; Simpson et al., 2006; Wolfe et al., 2010, 2015).

With FT, the frequency of each component in the SB is reduced by a fixed amount in Hertz, so the width of the DB is the same as the width of the SB; see the bottom-left panel of Figure 1. In most such systems, the DB is superimposed on the frequency components that are not lowered (those falling below the SB). Again, studies evaluating various FT systems have given mixed results. Improvements have been reported for hearing thresholds (Auriemmo et al., 2009), word-final /s, z/ detection (Robinson, Baer, & Moore, 2007), and consonant identification (Auriemmo et al., 2009; Kuk, Keenan, Korhonen, & Lau, 2009). However, other studies found no change in performance for word-final /s, z/ detection (Robinson, Stainsby, Baer, & Moore, 2009), or consonant identification (Robinson et al., 2007, 2009) and some studies found worse performance for identification of consonants in noise (Alexander, Kopun, & Stelmachowicz, 2014), and sentences in noise (Miller, Bates, & Brennan, 2016). Responses to questionnaires evaluating subjective outcomes showed either no preference (Miller et al., 2016) or a preference for conventional hearing aids over FT aids (Robinson et al., 2009).

**Figure 2.**
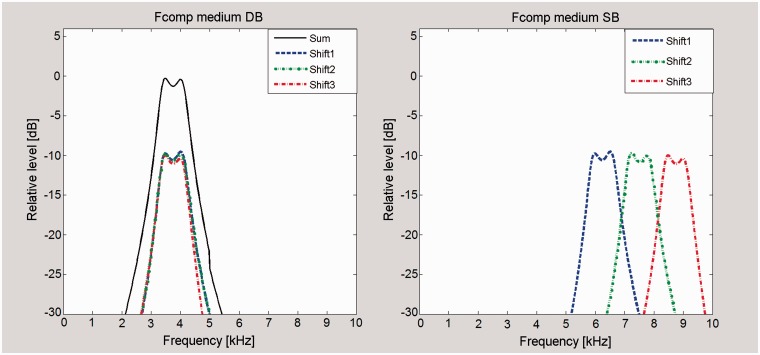
Example of the SB (right panel) and DB (left panel) for the medium setting of Fcomp. Each of the three subbands in the SB (termed “Shift1,” “Shift2,” and “Shift3” here) is transposed to the same DB. Therefore, the DB is narrower than the SB. Based on information provided by Bernafon AG. SB = source band; DB = destination band; Fcomp = frequency composition.

This article reports an evaluation of another form of FL that is implemented in commercial hearing aids. This is called frequency composition (Fcomp) by the manufacturer (Bernafon AG). The operation of Fcomp (Kuriger & Lesimple, 2012) is illustrated by the bottom-right panel in Figure 1 and is illustrated in more detail in Figure 2. The SB has three adjacent and equally wide subbands. Each of these subbands is transposed into a single DB whose bandwidth in Hertz equals that of each subband. Thus, information about spectral shape within the SB is partially lost, but information about the overall short-term energy within the SB and the temporal envelope within the SB is preserved. The relative level of the transposed components can be modified in the programming software. All other frequency components, including those originally falling within the DB, are preserved. Fcomp is enabled all the time, but its effects are usually only perceivable if the energy in the SB is greater than the energy in the DB. The calculations required for the implementation of Fcomp are made in parallel to the calculations that are needed for gain control and other functions, so the activation of Fcomp does not lead to any additional delay between the input and output of the hearing aid.

At the time of writing, there were no published evaluations of Fcomp. In addition, there is little published research evaluating FL for participants with diagnosed DRs. Glista et al. (2009), Glista, Scollie, and Sulkers (2012), and Ellis and Munro (2015b) did test their participants for DRs, but the settings of the FL were not influenced by the outcomes of the tests for DRs. To date, the study of Robinson et al. (2009) is the only one for which the settings of the FL were selected based on the characteristics of the DRs of the participants. For people with extensive high-frequency DRs, the available range for the DB may be rather limited. Fcomp allows the use of a relatively wide SB even when the DB is relatively narrow. This potentially allows provision of high-frequency cues in speech that would otherwise be inaudible or unusable. In contrast, both FC and FT have limitations in the width of the SB. For FT, the SB cannot be wider than the DB. For FC, a wide SB would require SF to be very low and the FC ratio to be high, both of which can impair sound quality and intelligibility (Alexander, 2016; Salorio-Corbetto, Baer, & Moore, 2017; Souza, Arehart, Kates, Croghan, & Gehani, 2013).

Most previous studies of FL have used experimental designs that might lead to confounding effects. In some studies, participants were trained only in the FL condition and not in the control condition (Korhonen & Kuk, 2008; Kuk et al., 2009), which could bias the outcomes (Füllgrabe, Baer, & Moore, 2010; Ling, 1968). For some studies, scores for the FL hearing aids were compared with those for unaided listening (Miller-Hansen, Nelson, Widen, & Simon, 2003) rather than to an appropriate control condition using hearing aids without FL. For some studies, the FL hearing aids were compared with the participants’ own hearing aids (Bohnert et al., 2010; Gifford, Dorman, Spahr, & McKarns, 2007; Miller-Hansen et al., 2003), which introduces confounds due to differences in microphones, receivers, amplitude compression, and fitting methods. Another common problem is the use of a fixed presentation order of the conditions, usually with the control condition first (Auriemmo et al., 2009; Gifford et al., 2007; Kuk et al., 2009), potentially biasing the outcomes toward the second condition tested, due to learning effects or, in the case of children, maturation effects (Auriemmo et al., 2009; Wolfe et al., 2011).

Some researchers have attempted to control for training effects using an “A-B-A” design, where A represents the control condition and B the condition with FL. The two sets of A scores were usually averaged (Ellis & Munro, 2015b; Glista et al., 2012; Simpson, Hersbach, et al., 2005; Simpson et al., 2006). However, there are problems with this design. The initial A scores may be adversely affected by lack of familiarity with the tasks, and the final A scores may be adversely affected by inappropriate use of cues learned during the more prolonged exposure to the FL condition, leading to systematic errors in the control condition. Some participants tested by Glista et al. (2012) showed these patterns. Some researchers tried to minimize order effects by using a hearing aid with each condition saved in a different program (Robinson et al., 2009). However, with this approach, frequent switching between programs could prevent participants from learning the novel cues provided by FL.

To avoid such confounding effects, we used a crossover design. There were two conditions, Fcomp-on and Fcomp-off, which had, respectively, Fcomp enabled and disabled, but were otherwise identical. Each participant wore the hearing aids using one condition for at least 9 weeks and then the other condition for at least 9 weeks. The order of conditions was counterbalanced across participants. Outcome measures were obtained at the end of each period of using a specific condition. A questionnaire to compare real-world performance with the two conditions was administered at the end of the study.

## Materials and Methods

### Participants

Ten adults (median age: 72 years, range: 66–85) with bilateral postlingual high-frequency sensorineural hearing loss completed the study. Participants were recruited from the laboratory database, which was developed using advertisements placed in hearing clinics, universities, and newsletters published by charities related to hearing loss. Table 1 shows demographic data for the participants. Audiometric inclusion criteria were as follows, having: (a) having sloping high-frequency sensorineural loss and (b) hearing thresholds within the recommended range for the hearing aids used (based on the hearing-aid datasheets), at least for frequencies up to 2 kHz, at least for one ear. Exclusion criteria were as follows: not being a native speaker of British English or having a language disability that could interfere with testing. Eight participants were male. Four participants had experience with FL hearing aids, although for two of them this experience was limited to a few listening sessions in the context of research projects. The two algorithms that the participants had used were as follows: (a) SoundRecover, which is a form of FC, either in the version implemented in commercial hearing aids or in modified versions with lower starting frequencies and (b) FT as described by Robinson et al. (2007) and (2009). Seven more participants were recruited but did not complete the trial for one of the following reasons: (a) They found the sound quality, management, or performance of the Fcomp hearing aids to be worse than for their own hearing aids in everyday life so they withdrew (*n* = 3), (b) Their hearing thresholds changed by more than 5 dB at any test frequency, as measured at the end of each test period (*n* = 2), and (c) They withdrew for personal reasons unrelated to the study (*n* = 2).

**Table 1.** Demographics of the Participants Including Age, Etiology or Risk Factors for Hearing Loss, Duration of Their Hearing Loss, Duration of Hearing-Aid Use, Type of Hearing Aid Used Prior to the Trial (Own HA), Previous Experience With Frequency-Lowering, and Trial Hearing-Aid Model (Robinson et al., 2007, 2009).

ID	Age (years)	Etiology or risk factors	Duration (years)	Hearing-aid use (years)	Own HA	Experience with frequency-lowering	Evaluation model
P01	72	Unknown	8	8	R: Oticon Delta; L: Phonak Audéo	SoundRecover (O-M) and CT	Nano RITE (PW)
P03	75	Family history	35	31	Oticon Spirit Zest P	SoundRecover (M) and CT	Compact Plus
P04	68	Noise exposure	14	4	Oticon Spirit Zest	No experience	Nano RITE (ST)
P05	72	Ototoxicity	14	4	Specsavers 430	No experience	Nano RITE (ST)
P06	72	Noise exposure	41	2	Phonak Exélia Art P	Lab experience (SoundRecover M and CT)	Nano RITE (R = ST; L = PW)
P07	85	Ototoxicity	12	7	Oticon Spirit Zest	No experience	Nano RITE (ST)
P09	66	Unknown	31	30	Oticon Spirit Zest P	Lab experience (SoundRecover M and CT)	Compact Plus
P10	71	Unknown	6	6	Siemens Reflex Air	No experience	Nano RITE (ST)
P13	80	Noise exposure	24	3	Oticon Spirit Zest P	No experience	Nano RITE (R = ST; L = PW)
P14	69	Age related	10	0	N/A	No experience	Nano RITE (ST)

*Note.* R = right; L = left; PW = power receiver; ST = standard receiver; SoundRecover O = original version of SoundRecover; Sound Recover M = modified SoundRecover; CT = Cambridge transposition.

The research was approved by the Cambridge Research Ethics Committee (reference number 11/H0306/2). Written consent was obtained from all participants.

### Basic Hearing Assessment

**Figure 3.**
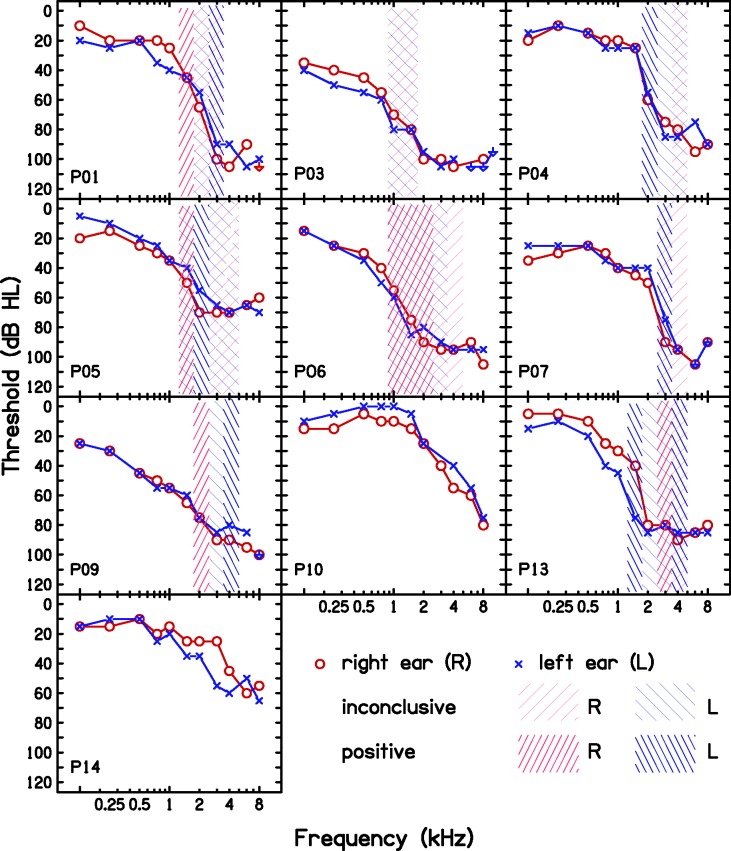
Audiograms of the participants. The shaded areas show the outcomes of the TEN(HL) test.

Audiometry was performed using a Grason-Stadler GSI-61 audiometer. Pure-tone thresholds were obtained at octave and semioctave frequencies from 0.125 to 8 kHz for air conduction using ER-3A insert earphones, and at octave frequencies between 0.25 and 4 kHz for bone conduction. Figure 3 shows the audiograms of the participants.

### Detection and Characterization of DRs

**Figure 4.**
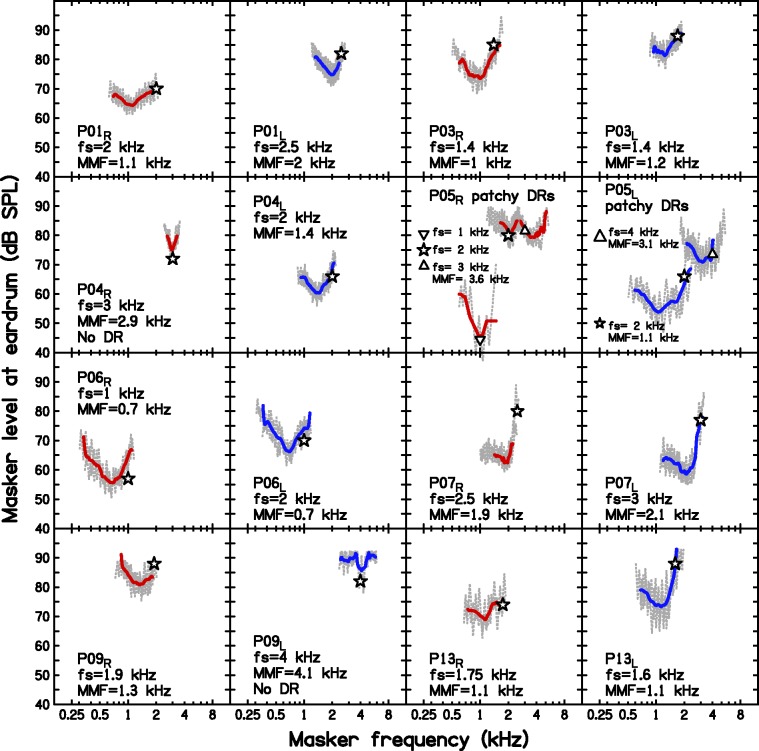
Fast PTCs obtained for the participants for whom the TEN(HL) test outcome was inconclusive or positive. The open symbols indicate the level and frequency of the signal. The jagged line shows the masker levels visited. The continuous line shows the combination of an upward sweep and a downward sweep in masker center frequency (except for P05_R_, *f*_s _= 1 kHz and P09_L_, for whom only upward sweeps were available) after smoothing each of them. All fast PTCs show a significantly shifted tip, which indicates a DR, except for P04_R_, P05_R_ *f_s_* = 1 and 2 kHz, and P09_L_. The MMF is given only when it is shifted by more than 10% from *f_s_*. MMF = minimum masker frequency; *f*s = frequency signal; DR = dead region.

DRs were diagnosed and characterized following the procedures and using the equipment described by Salorio-Corbetto et al. (2017). A high-frequency (basal) DR is characterized by the value of the characteristic frequency of the inner hair cells or neurons immediately below the DR, which is called the edge frequency, *f_e_* (Moore, 2001). The values of *f_e_* were roughly estimated using the TEN(HL) test (Moore, Glasberg, & Stone, 2004) and then estimated more precisely using fast psychophysical tuning curves (PTCs; Sek & Moore, 2011). A DR was deemed to be present if the frequency at the tip of the fast PTC (termed the minimum masker frequency, MMF) was shifted relative to the frequency of the signal (*f*_s_) by 10% or more (Moore & Malicka, 2013). In these cases, the MMF was taken as the estimate of *f_e_*. Figure 3 shows the TEN(HL)-test outcomes, and Figure 4 shows the fast PTCs and MMF values. Five participants had extensive DRs bilaterally (P01, P03, P06, P07, and P13), two participants had unilateral DRs (P04 and P09), one had patchy DRs (P05), and two had no DRs (P10, P14).

### Duration of the Evaluation Period

A single-blind two-period, two-condition (Fcomp-on or Fcomp-off) crossover design was used. The participants were not told which condition was being tested during each period to avoid this knowledge affecting the outcome measures (Dawes, Hopkins, & Munro, 2013), although some reported that they could hear the effect of the Fcomp processing. The order of conditions was determined randomly for each participant with the constraint that the two possible orders were used equally. Each period of aid use lasted for 9.7 to 31 weeks (median 18.2 weeks), depending on the availability of the participant. Effort was put into keeping the duration of the two periods as similar as possible for each participant. Outcome measures were obtained toward the end of each period, except for the questionnaire, which was administered after both periods were completed.

### Hearing Aids and Hearing-Aid Fitting

Bernafon Acriva 9 Compact Plus (AR9 CPx) or Nano RITE (AR9 NR) behind-the-ear (BTE) digital hearing aids with either standard or power receivers were used. The hearing-aid model and receiver were chosen for each participant according to the gain requirements imposed by the hearing loss (see Table 1). The frequency ranges of the hearing aids were between 0.1 and 6 kHz (Compact Plus BTE) or 0.1 and about 6.9 kHz (Nano RITE BTE). The hearing aids use “ChannelFree”™ amplification, in which amplitude compression is implemented by a single time-varying digital filter whose coefficients are changed dynamically, up to 20,000 times per second (Bernafon, 2009; Plyler, Reber, Kovach, Galloway, & Humphrey, 2013; Schaub, 2010). Additional features included adaptive directionality, feedback cancellation, noise reduction, transient noise reduction, binaural coordination of gains, optional telecoil program, data logging, and “learning” volume control. The last feature was disabled to keep the gain settings stable across conditions. A remote control or streamer was used to select programs. Each hearing aid had three programs: (a) *Multi-environment*, (b) *Noisy situations*, (c) *Telecoil* that kept the microphone of the hearing aid active but with a 6-dB gain reduction. For participant P09, the telecoil program was set to “loop only,” as she used this program in church and found the voices of children to be very distracting. This meant that Fcomp was never active when she used the telecoil program, although that program was not used frequently.

Real-ear aided gain targets calculated using the CAM2A method (Moore, Glasberg, & Stone, 2010) were matched as closely as possible using an Interacoustics Affinity real-ear measurement system. The stimulus used for verification was a speech-shaped noise with a level of 50, 65, and 80 dB SPL. All the advanced features of the hearing aids were switched off during these measurements. The gains were kept constant after the first fitting, except for some participants for whom it was necessary to modify the gains to ensure listening comfort. P03 requested an increase in volume after the first week of wearing the hearing aids. A gain increase of 2 dB at all frequencies was implemented. For P04, the gains were decreased 2 days after fitting, by 2 dB at 2 and 3 kHz, and by 3 dB at 4 and 6 kHz. Nine weeks later, an additional overall reduction of 3 dB for the right hearing aid and 5 dB for the left hearing aid was implemented to avoid the need for him to manually adjust the volume each time he switched the hearing aids on. For P07, a 3-dB reduction of gain at all frequencies was implemented 1 week after fitting. For P09, a 3-dB reduction of gain at 3 and 4 kHz was implemented 1 week after fitting.

For Fcomp, there are three possible SBs (3.8–7.2 kHz, 5.3–9.6 kHz, 6.5–10 kHz), each linked to a different DB (1.5–2.9 kHz, 2.9–4.6 kHz, 4.6–6.5 kHz, respectively). The edge frequencies are defined as the −15-dB points. The manufacturer recommends that the center frequencies of the SB and DB should be reduced as the severity of high-frequency loss increases. We started by following this recommendation. The audibility achieved in this way was assessed by estimating the center frequency of the 1/3-octave band in which the root-mean-square (RMS) output of the hearing-aid intersected the hearing threshold (*Thr_f_*); see later for details. If *Thr_f_* was close to or below the lower edge of the DB or if the DB fell well within a DR, a lower range was used if available, provided that the participant did not report poor sound quality of his or her own voice or the experimenter’s voice. The default combinations of SB and DB were used for most participants but (P07) was fitted with a lower SB or DB combination. The manufacturer would not recommend the use of Fcomp for P05, P10, P14, but nevertheless it was activated for them. It should be noted that the setting with the highest SB and DB was not used here, as the DB was outside the range of audibility of the participants.

A parameter in the fitting software determines the relative level of the transposed components. The settings are called *weak*, *medium*, and *strong*, and each differs from its neighbor by 6 dB in level. This parameter was initially set to *strong* for all participants, but one-step reductions were made for P05 and P09 because they complained about the sound quality of their own voice or the voices of others. Table 2 shows the Fcomp settings for each participant.

**Table 2.** Fcomp Settings and Estimated Audibility Measures for Each Participant.

			Center frequency of Verifit band (kHz)		
ID	*Thr_f_* (kHz)	DB (kHz)	4	5	6.3
P01 L	2.35	1.5–2.9	Y	Y	Y
P03 L	1.31	1.5–2.9	N	N	N
P04 R	2.86	2.9–4.6	NL	N	N
P05 L	3.08	1.5–2.9	Y	Y	N
P06 L	1.85	1.5–2.9	N	N	N
P07 L	2.43	1.5–2.9	Y	Y	Y
P09 L	1.98	1.5–2.9	N	N	N
P10 R	4.71	2.9–4.6	NL	Y	Y
P13 R	1.98	1.5–2.9	Y	Y	Y
P14 R	3.91	2.9–4.6	NL	Y	Y
P14 L	3.51	2.9–4.6	NL	N	N
Average	2.72				

*Note.* *Thr_f_* indicates the center frequency of the 1/3-octave band for which the root-mean-square output of the hearing-aid intersected the hearing threshold. DB indicates the range of the destination band. “Y” indicates that the corresponding Verifit band was deemed to be 50% or more audible. “N” indicates that audibility was poor or the band was not audible. “NL” indicates that the band was not lowered, as it was outside the SB. For P14, estimated audibility is shown for both ears because some tests were done only for one ear.

### Outcome Measures

#### Estimation of audibility

The audibility of the frequency-lowered sounds was estimated using the verification signals implemented in the Audioscan Verifit, as suggested by the manufacturer of the hearing aids. These signals are passages of speech that are filtered so as to have 30-dB attenuation for frequencies above 1 kHz, except for a 1/3-octave wide band with a center frequency of 3.15, 4, 5, or 6.3 kHz. This allowed the transposed components in the output of the hearing aid to be identified easily. Note that, for the two SBs that were used, each subband within the SB had a width slightly less than 1/3 octave. Measurements were taken in real ears. A band was deemed to be audible if its RMS level was at or above the hearing threshold at the relevant center frequency.

#### Consonant identification: The vowel-consonant-vowel test

For all speech tests, the participant sat in a sound-attenuating booth, at a distance of 1 m from one or two Tannoy Precision 8D self-powered loudspeakers connected to a Samsung P510 laptop with an external M-Audio Audiophile USB soundcard. Presentation levels were measured with a Lucas CEL-414 precision impulse sound level meter at the approximate position of the center of the head of the participant.

The vowel-consonant-vowel (VCV) test (Robinson et al., 2007, 2009) is a closed-set consonant identification test that uses VCV combinations made of 1 of 21 consonants (/p, t, k, b, d, g, f, h, s, ʃ, ɵ, v, z, dʒ, tʃ, l, r, w, y, m, n/) and one of three vowels (/a, i, u/) uttered by a single female talker. The initial and final vowels are the same. Each test list contains 63 items. Presentation was via a single loudspeaker at 0° azimuth. A computer screen was placed in front of the participant. Participants responded verbally, and the examiner, who was in a corner of the same booth, away from the participant and the loudspeaker, entered the responses into the computer. The presentation level was 65 dB SPL, except for P10 and P14, for whom the level was set to 55 dB SPL to avoid ceiling effects. In addition, P14 did not wear the hearing aid in his better-hearing ear (right), and this was plugged with a foam plug, again to avoid ceiling effects. The session started with a VCV list for which feedback was provided. The score for this list was not taken into account. Subsequent lists were presented without feedback. Six lists were used for each participant and for each condition.

#### Word-final /s, z/ detection: The S-test

The S-test, developed by Robinson et al. (2007), measures the ability to detect word-final /s, z/. The speaker was a native speaker of British English. In each trial, a pair of words differing only in the presence of word-final /s, z/ (e.g., *book* or *books; pig* or *pigs*) was presented on a screen but only one word was played. The task was to identify the word that was played. The presentation level was 65 dB SPL except for P10 and P14, for whom the presentation level was reduced to 55 dB SPL to avoid ceiling effects. Presentation was via a single loudspeaker at 0° azimuth. Participants gave an oral response, and this was recorded by the experimenter using a MATLAB-controlled interface.

#### Intelligibility of speech in background noise

The target stimuli were sentences from the adaptive sentence list corpus (MacLeod & Summerfield, 1990) spoken by a male native speaker of British English. Each list consisted of 15 sentences. Examples of the sentences are as follows: “They moved the furniture” and “The towel dripped on the carpet.” Participants wrote down their response for each sentence. Each sentence had three keywords. A word was scored as correct if it was exactly right, or if the number of syllables was not reduced but the wrong tense was used (e.g., *working* instead of *work*), or if the word was phonetically correct (e.g., *too* instead of *two*). Spelling mistakes and plural distinctions were ignored. Words were marked as incorrect if the word was completely different, the gender was incorrect (e.g., *he* or *she*), or the target word was put into a longer word with a different meaning (e.g., *yellow* used instead of *yell*).

The long-term spectrum of the speech was shaped to match the long-term average spectrum estimated by Moore et al. (2008). This was done in MATLAB using a 45-tap finite-impulse-response filter. The background was two-talker babble, selected because it provides opportunities to “listen in the dips.” The two talkers used to produce the babble were taken from recordings described by Moore et al. (2008). Both were male native speakers of British English. The spectrum of the babble was shaped to match the long-term spectrum of the speech.

Participants sat in a soundproof booth, with both hearing aids turned on, set to their preferred volume. The target sentences and babble were either colocated or spatially separated. Presentation was via two loudspeakers at azimuths of 60° and −60°. The participant looked to the front (0° azimuth) during the test. The soundcard output was routed to the loudspeakers via Tucker Davis PA4 programmable attenuators and Tucker Davis SM3 mixers. For the colocated case, the target speech and babble were played out either from the right loudspeaker or from the left loudspeaker. For the spatially separated case, the target speech was played out from one loudspeaker, and the babble was played out from the other one. This gave four combinations of talker and masker locations (both from the right, both from the left, talker from right and masker from left, talker from left and masker from right) whose order was randomized across participants. Each sentence was presented with a randomly selected segment of the background babble. The babble started 500 ms before the sentence and ended 500 ms after the sentence. The level of the speech was fixed at 65 dB (A). The signal-to-babble ratio (SBR) was selected for each participant, based on practice runs, so as to achieve mid-range performance. This was done separately for each spatial configuration. For each spatial configuration, two lists of practice sentences were presented, followed by two test lists. Practice lists were drawn from the BKB corpus (Bench & Bamford, 1979). Each list consisted of 16 sentences. They were spoken by the same speaker as for the test lists, and the masker was the same as for the test lists. Initially the practice lists were presented at 4 dB SBR for the colocated condition and −6 SBR for the spatially separated condition. If performance was close to ceiling or floor, the SBR vas varied to find a SBR that led to mid-level performance. Once the SBR was selected for each condition, it was maintained throughout the evaluation period.

#### Speech, spatial, and qualities of hearing scale

The Speech, Spatial, and Qualities of Hearing Scale in its comparison version (SSQ-C; Gatehouse & Noble, 2004) was used to assess whether the participants experienced differences between the two conditions in everyday life. The SSQ has three subscales: (a) speech hearing, based on 14 items whose aim is to assess speech communication in different settings, (b) spatial hearing, based on 17 items assessing the ability of the hearing-aid user to make judgments of sound distance and direction and to discriminate movement, and (c) other qualities, based on 18 items related to segregation of sounds, clarity and naturalness, and listening effort.

The SSQ-C is designed to compare experience with two hearing aids worn in succession. After the two periods of the trial were completed, participants were asked to complete the questionnaire at home. They were given the chance to discuss any concerns. Although an interview format is preferred to self-administration (Gatehouse & Noble, 2004; Singh & Kathleen Pichora-Fuller, 2010), we chose the latter because most participants would have been unable to commit to an additional session to complete the questionnaire.

## Results

### Estimated Audibility

Table 2 shows the values of *Thr_f_*, the frequency at which the RMS level of the speech intersected the hearing threshold, measured with Fcomp-off. The table also shows the DB and whether a given SB was audible after FL (“Y,” yes or “N,” no). “NL” mean that the band was not lowered, as it was not included in the SB. The outcomes for the ear with best estimated audibility are shown, except for P14, for whom the outcomes for both ears are shown, as he was tested with the left ear only for the VCVs. Fcomp improved audibility for 6 out of the 10 participants. Little or no audibility improvement was estimated for four participants (P03, P04, P06, and P09).

### Consonant Identification

**Figure 5.**
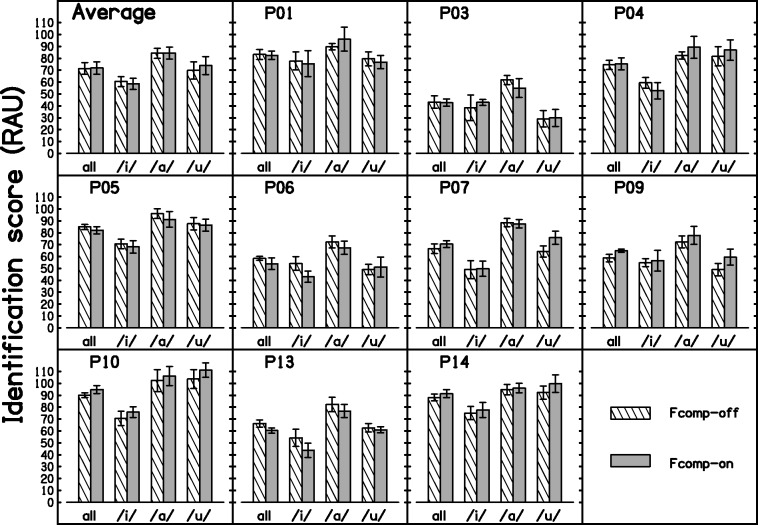
Average and individual scores for the VCV test expressed in RAU, plotted for all vowel contexts combined (“all”) and separately for each vowel context (/i/, /a/, /u/). Error bars show ± 1 standard error. Fcomp = frequency composition.

Figure 5 shows the percent correct scores for the group and for each participant, for the two test conditions. Results are shown for each vowel context separately and for all contexts combined. Percent correct scores were transformed to rationalized arcsine units (RAU) in order to satisfy the assumptions of analysis of variance (ANOVA). After transformation, the scores were corrected for guessing using corrections suggested by Sherbecoe and Studebaker (2004). The two conditions led to similar overall outcomes. Mean consonant-identification scores were 71.5 and 72.3 RAU with Fcomp-off and Fcomp-on, respectively. A two-way repeated measures ANOVA with factors condition and vowel context showed that the effect of condition was not significant, *F*(1, 9) = 0.32, *p* = .583. The effect of vowel context was highly significant, *F*(2, 18) = 24.54, *p* < .001. Post hoc analyses with Bonferroni correction indicated that performance for each of the three vowel contexts was significantly different from that for each of the other contexts, /a/ leading to the highest scores, and /i/ the lowest scores. There was a significant interaction between condition and vowel context, *F*(2, 18) = 4.45, *p* = .027. While there was almost no difference between the two conditions when the vowel context was /i/or /a/ (−1.83 and 0.08-RAU differences, respectively), the mean score was 4 RAU higher with Fcomp-on than with Fcomp-off when the vowel context was /u/. A separate one-way ANOVA on the scores for each vowel was performed. For the vowel contexts /a/ and /i/, the effect of condition was not significant, *F*(1, 9) = 0, *p* = .967, and *F*(1, 9) = 0.95, *p* = .356. For the vowel context /u/, there was a marginally significant effect of condition, *F*(1, 9) = 5.90, *p* = .038. However, the effect was deemed not significant after correcting the *p* value to allow for the fact that three ANOVAs were performed, one for each vowel context (*p* = .114).

#### Consonant confusions and transmission of phonetic-feature information

**Figure 6.**
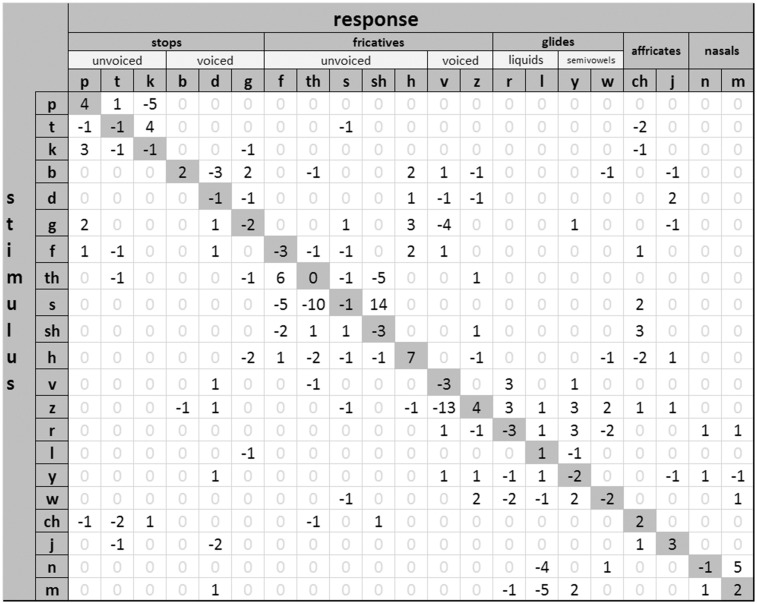
Consonant-confusion matrix showing the difference in responses for Fcomp-on and Fcomp-off. All numbers represent percentage points. Positive numbers on the diagonal mean that Fcomp improved the identification score for that consonant, while negative numbers mean that Fcomp worsened the score. Positive numbers off the diagonal mean that confusions between the two consonants determining the cell were increased when Fcomp was used, while negative numbers mean that confusions decreased. /θ/, /ʃ/, /tʃ/, and /tʒ/ are labeled using their orthographic representations,* th, sh, ch, and j*, respectively.

Fcomp may change the patterns of consonant confusions while having little or no effect on the overall score. To explore this, consonant-confusion matrices were calculated from the results of the VCV test. Figure 6 shows a difference matrix obtained by subtracting the confusion matrix for condition Fcomp-off from that for condition Fcomp-on. Average changes in consonant confusions were small, although the individual results showed greater changes in the confusion patterns. For the group, the most salient changes with Fcomp-on were that /s/ was confused with /ʃ/ rather than /f/ or /ɵ/, and /ɵ/ was confused with /f/ rather than /s/ and /ʃ/. The confusion of /z/ with /v/ was reduced with Fcomp-on, but confusions of /z/ with glides and affricates increased, giving only a small overall change in the identification of /z/.

“Sequential Information Feature Analysis” (SINFA; Wang & Bilger, 1973) was performed to clarify the effect of condition on the transmission of the articulatory features voicing (voiced or unvoiced), manner (stop, fricative, approximant, affricate, and nasal), and place (bilabial, labiodental, dental, alveolar, palatoalveolar, palatal, glottal, and velar). A feature matrix (Table 3) was specified for each of the 21 consonants used in the VCV test. The confusion matrices were analyzed using the software FIX, developed by Mike Johnson (Department of Phonetics, University College London) and available at ftp://ftp.phon.ucl.ac.uk/pub/fix/. SINFA allows determination of the contribution of each of a selected set of features to the total information transmitted. An iterative process is used to obtain the independent contribution of each feature to the transmission of information. For each iteration, the features carrying most of the information in previous iterations are held constant. FIX returns three results: the total information transmitted (in bits of information), the proportion of information transmitted by each of the selected features relative to the input, and the proportion of information transmitted relative to the total information transmitted. Here we focused on the first two. SINFA was carried out for each condition separately, for each participant and for the group as a whole. When performing SINFA, if too many features are used at the same time, the information transmitted for the features that carry less information (and therefore are analyzed later in the process) may not be reported. To avoid this situation, SINFA analyses were performed separately for two submatrices obtained from Table 3. One submatrix contained the features of place of articulation, and the other contained the features of manner of articulation. The feature of voicing, which is robust and largely unaffected by FL, was added to the smallest of the matrices, the manner matrix.

**Table 3.** Feature Matrix Used for SINFA.

	p	t	k	b	d	g	f	ɵ	s	ʃ	h	v	z	r	l	y	w	tʃ	dʒ	n	m
bil	1	0	0	1	0	0	0	0	0	0	0	0	0	0	0	0	1	0	0	0	1
ld	0	0	0	0	0	0	1	0	0	0	0	1	0	0	0	0	0	0	0	0	0
den	0	0	0	0	0	0	0	1	0	0	0	0	0	0	0	0	0	0	0	0	0
alv	0	1	0	0	1	0	0	0	1	0	0	0	1	1	1	0	0	0	0	1	0
palv	0	0	0	0	0	0	0	0	0	1	0	0	0	0	0	0	0	1	1	0	0
pal	0	0	0	0	0	0	0	0	0	0	0	0	0	0	0	1	0	0	0	0	0
glo	0	0	0	0	0	0	0	0	0	0	1	0	0	0	0	0	0	0	0	0	0
vel	0	0	1	0	0	1	0	0	0	0	0	0	0	0	0	0	0	0	0	0	0
stop	1	1	1	1	1	1	0	0	0	0	0	0	0	0	0	0	0	0	0	0	0
fric	0	0	0	0	0	0	1	1	1	1	1	1	1	0	0	0	0	0	0	0	0
appr	0	0	0	0	0	0	0	0	0	0	0	0	0	1	1	1	1	0	0	0	0
affr	0	0	0	0	0	0	0	0	0	0	0	0	0	0	0	0	0	1	1	0	0
nas	0	0	0	0	0	0	0	0	0	0	0	0	0	0	0	0	0	0	0	1	1
voic	0	0	0	1	1	1	0	0	0	0	0	1	1	1	1	1	1	0	1	1	1

*Note.* bil = bilabial; ld = labiodental; den = dental; alv = alveolar; palv = palatoalveolar; pal = palatal; glo = glottal; vel = velar; fric = fricative; appr = approximant; affr = affricate; nas = nasal; voic = voicing. The leftmost column shows the features used for the analyses. The top row shows the consonants included in the stimuli.

**Figure 7.**
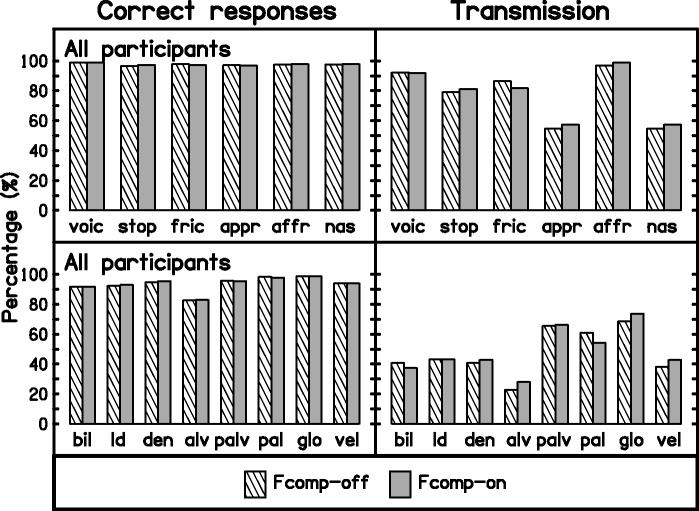
Outcomes of SINFA for the sum of responses of the participants. Correct responses are shown in the left panels, and the percentage of information transmitted for each feature is shown in the right panels. Analyses were made for the features voicing and manner of articulation (top panels) and for the feature of place of articulation (bottom panels). The label used for each feature is specified in Table 3. Fcomp = frequency composition.

Figure 7 shows the outcomes of the analyses for the whole group, with percent correct for each feature on the left and percent information transmitted (relative to the information in the stimuli) on the right. Differences across the two conditions were very small. The total information transmitted was 3.18 bits for Fcomp-off and 3.17 bits for Fcomp-on. For individual participants, there were some differences in the transmission of features across conditions. These differences tended to balance each other, giving only small differences in the total information transmitted (Table 4). The largest differences occurred for P07, P10, and P13. For P07 and P10, the total information transmitted was slightly greater for Fcomp-on than for Fcomp-off. For P07, the difference arose mainly from increases in the transmission of the affricate manner of articulation and for most places of articulation, especially the palatoalveolar and palatal places. For P10, information transmission was lower for Fcomp-on for the fricative and affricate manners of articulation but was higher for Fcomp-on for the bilabial, labiodental, and dental places of articulation. For P13, information transmission was lower for Fcomp-on for manner of articulation and some places of articulation (especially for dental and alveolar places of articulation).

**Table 4.** Total Information Transmitted for Each Participant and Condition and Differences Across Conditions.

ID	Fcomp-off	Fcomp-on	Fcomp-on − Fcomp-off
P01	3.757	3.781	0.024
P03	2.56	2.593	0.033
P04	3.591	3.536	−0.055
P05	3.841	3.778	−0.063
P06	3.161	3.077	−0.084
P07	3.346	3.482	0.136
P09	3.07	3.162	0.092
P10	3.964	4.073	0.109
P13	3.387	3.122	−0.265
P14	3.998	4.017	0.019
Mean	3.184	3.174	−0.01

*Note.* Fcomp = frequency composition.

### Word-Final /s, z/ Detection

**Figure 8.**
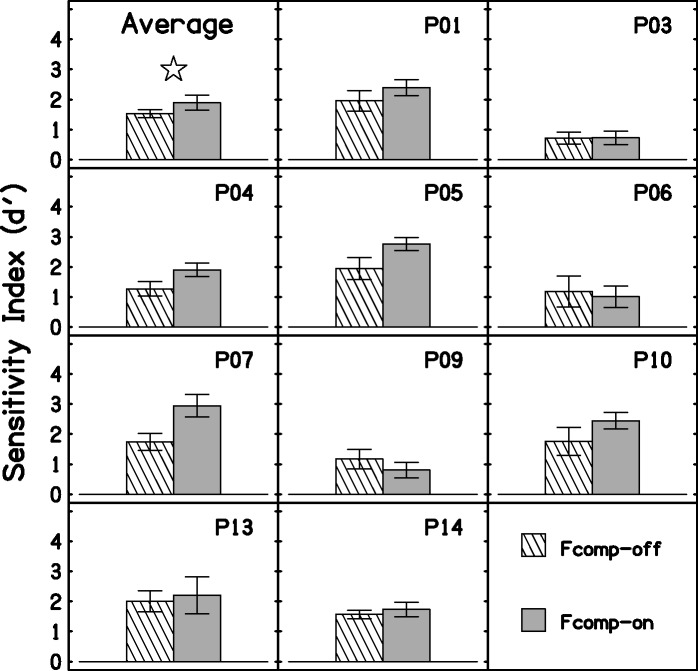
Average and individual scores for the S-test. Error bars show ± 1 standard error. The star denotes a significant difference.

Both *hits* (correct detections of /s, z/) and *false alarms* (reporting that /s, z/ was present when this was not the case) were counted. These were used to calculate *d*′, the sensitivity index based on signal detection theory (McNicol, 2004; Moore, 2012). The log-linear rule (Hautus, 1995) was used to minimize bias of the estimated values of *d*′ when there were 100% hits or zero false alarms. With this rule, 1 is added to the number of trials, and 0.5 is added to the number of hits and false alarms. For example, if the number of hits is 24/24 (100%), the revised value is 24.5/25. Figure 8 shows the *d*′ scores for the group and for each participant. On average, Fcomp-on led to better detection of word-final /s, z/ than Fcomp-off. The effect was significant for the group, *F*(1, 9) = 5.64, *p* = .042, η_p_^2 ^= 0.39. Five participants performed better with Fcomp-on (P01, P04, P05, P07, P10), while one participant (P09) performed slightly worse. The remaining four participants achieved similar scores across conditions.

### Identification of Speech in Noise

**Figure 9.**
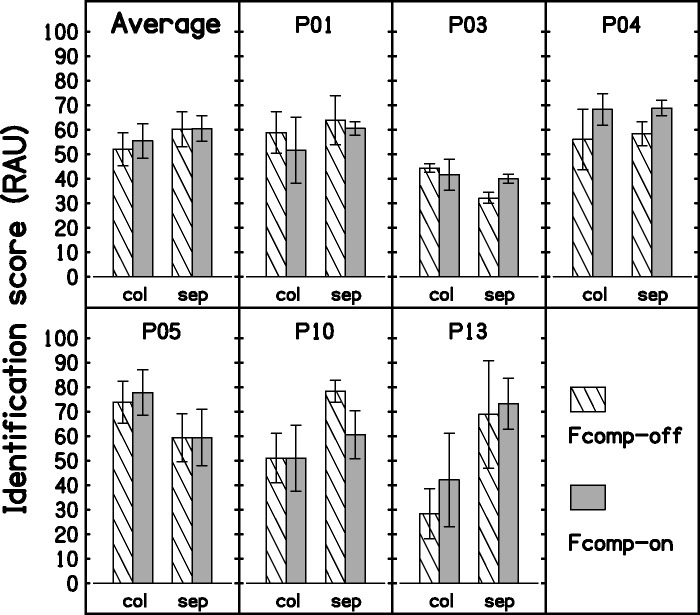
Mean and individual outcomes for the speech-in-noise test. Results in RAU are plotted separately for the colocated (“col”) and the spatially separated (“sep”) configurations. Error bars show ± 1 standard error.

Only six participants completed this test because of limited availability. Fcomp provided audibility improvement, as measured using the Verifit, for four of these, but provided little improvement for the other two. Figure 9 shows the outcomes expressed as percent correct. Scores were transformed into RAU, and a correction to account for the number of keywords in each list was applied before statistical analyses. For the colocated masker, a further transformation (square root) was needed to achieve a normal distribution. However, the outcome of the statistical analysis was essentially the same when this transformation was omitted. A one-way ANOVA with factor condition was performed on the scores for each spatial configuration. The difference between conditions was not significant, *F*(1, 5) = 0.87, *p* = .394, for the colocated configuration, and *F*(1, 5) = 0, *p* = .991, for the spatially separated configuration. Three participants showed differences across conditions of 10% or more for at least one spatial configuration, but these differences may have been related to the order of testing of the conditions.

### Speech, Spatial, and Qualities of Hearing Scale

**Figure 10.**
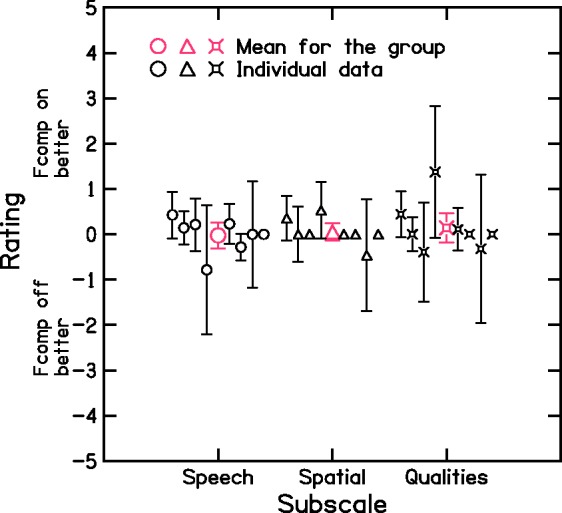
Outcomes of the SSQ-C questionnaire. Positive values indicate better scores for Fcomp-on, and negative values indicate better scores for Fcomp-off. Values close to zero indicate no difference. Average group outcomes are plotted in red, and individual outcomes are plotted in black. Fcomp = frequency composition.

Eight participants completed the SSQ-C. In the SSQ-C, a score of 0 means that there was no difference between conditions. A positive score means that performance with the condition used at the time of responding was better than with the condition used previously. Thus, for the participants who were tested first with Fcomp-off, a positive score meant that Fcomp-on was judged as better. The opposite is true for the participants who were tested first with Fcomp-on. For the latter, the signs of the scores were reversed before averaging the data across participants. Thus, for the data shown in Figure 10, a positive number indicates a better score with Fcomp-on, and a negative number indicates a better score with Fcomp-off. Scores were averaged for each subscale and each participant (black lines and symbols) and for the group (red lines and symbols).

For each of the three subscales, mean scores for the group were close to 0. This was also the case for most individual participants. However, for some participants, scores were better for one of the two conditions only for some questions in each subscale. Sometimes the direction of the difference between conditions was not consistent within a given scale. This explains the fact that the individual standard deviations were large in some cases. For example, for the speech subscale, P05 rated Fcomp-off as better (with scores of −2 or −3) for most situations involving interaction with a group or communication partner, or when a competing talker was present, but she reported no difference or Fcomp-on being slightly better for other situations. Similarly, P13 rated Fcomp-off as being better (with scores of −1.2 to −1.8) in conversational situations with competing speakers but rated Fcomp-on as better when listening to speech in places where there was reverberation (score of 2.2). For the spatial subscale, P13 rated Fcomp-on as being better for localization of a lawnmower outdoors and for the externalization of sounds (scores of 2 and 2.2, respectively), but he rated Fcomp-off as better for localizing a dog barking (score of −2), for estimating the direction of the displacement of a moving source (score of −2.35), and for estimating the distance of a source (scores ranging from −1.2 to −2). For the qualities of speech subscale, P05 rated Fcomp-on as better (scores of 2 or 3) for the sound quality of everyday sounds, her own voice and the voices of others, while showing no clear difference across conditions in other situations. P13 reported that with Fcomp-on environmental sounds seemed more separate from one another and he needed to concentrate less and put less effort into speech understanding (scores of 2.4 for environmental sounds and 2.0 and 1.8 for understanding speech). However, he rated Fcomp-off as better when listening to music and judging the naturalness of sounds (scores between −2.1 and −3.1, and −1.9, respectively). Similarly, P04, who is musically trained, reported better sound quality with Fcomp-off for music, naturalness of own voice and environmental sounds (score of −2.0) with no difference between conditions in other situations. It is worth mentioning that in normal usage, problems with music are unlikely to arise, as by default Fcomp is switched off for the music program.

## Discussion

We evaluated Fcomp with a single-blind crossover design using a control condition for which Fcomp was switched off. Ten participants with high-frequency hearing loss completed the study. Participants were not informed of the condition being tested, but some of them (P01, P04, P07, P09, P14) reported that they could hear the effect of Fcomp.

The estimated audibility of the high-frequency components of speech improved for 6 out of 10 participants. For P04, estimated audibility was not improved by the use of Fcomp but he nevertheless showed better detection of word-final /s, z/ with Fcomp-on and reported worse sound quality for music in the SSQ-C questionnaire with Fcomp-on. This suggests that the stimuli used to estimate audibility were not ideal. Audibility with Fcomp-on could have been underestimated because (a) the width of the high-frequency bands used in the Verifit system is smaller than the bandwidth of fricative or affricate consonants such as /s/ and /dʒ/, (b) Fcomp was effectively active only occasionally during the presentation of the stimuli, as strong high-frequency components in speech are not present all of the time, (c) the Verifit bands have a width that is slightly greater than the width of each of the SB subbands. When using natural speech, the stops, fricatives, and affricates will simultaneously produce inputs from more than one subband, and often from all three, which would lead to greater audibility than estimated by the Verifit system.

It seems likely that the increase in audibility provided by Fcomp was underestimated in our measurements and that an audibility increase was provided for almost all of the participants. However, participant P03 had a value of *Thr_f_* that was below the lower edge of the DB. This means that for sounds within the DB, audibility was probably very poor or zero. To assess whether this affected the study outcomes, we repeated all of the statistical analyses excluding the results for P03. This did not lead to any changes in the significance of any effects or interactions.

A reliable method of estimating the audibility of the FL sounds may help to avoid extreme settings of FL in attempts to reach audibility (Miller et al., 2016) and may also help to avoid weak settings that do not increase access to high-frequency sounds. Stimuli consisting of simulated /s/ and /ʃ/ sounds have been proposed for this purpose (Scollie et al., 2016).

Fcomp did not significantly improve overall consonant identification. There was a significant effect of vowel context, with context /a/ leading to the highest scores, and /i/ to the lowest scores. This effect is most likely related to the use of formant transitions as a cue for consonant identification. The vowel /i/ has second and third formants that are higher in frequency than those for /a/ and /u/ (Peterson & Barney, 1952). Hearing loss at high frequencies could adversely affect the use of formant transition information by reducing audibility or impairing the spectral and temporal analysis mechanisms used to extract this information. There was a significant interaction between condition and vowel context. There was a trend for the difference between Fcomp-on and Fcomp-off to be greater for the vowel context /u/. However, when a separate ANOVA with factor condition was performed for the vowel context /u/, the effect was deemed not significant after correcting for multiple comparisons.

There was a trend for confusions of /s/ with /ʃ/ to increase and for confusions of /s/ with /f/ to decrease with Fcomp-on. This may be explained by changes in the audibility of the frication noise of /s/. Listeners tend to identify high amplitude ratios between the frication noise and the vowel in the F3 region as /ʃ/ rather than /s/ (Hedrick & Ohde, 1993). Also, hearing-impaired listeners rely on the amplitude-ratio cue to a greater extent than normal-hearing listeners, who use both spectral cues and relative-amplitude cues (Hedrick & Jesteadt, 1996; Hedrick, Schulte, & Jesteadt, 1995). For our participants, spectral analysis is likely to have been poor, especially for those with extensive DRs, probably making them more reliant on relative amplitude cues. If Fcomp made the frication noise more audible, then the effective noise-vowel ratio would have increased, leading the participants to label /s/ as /ʃ/. A similar explanation may account for the finding that, with Fcomp-off, /s/ was often identified as /f/, whose frication noise is weaker than that of /s/ (Jongman et al., 2000). It is possible that fine tuning the gain applied to the transposed sound would decrease /s, ʃ/ confusions, but that was not attempted here. It is also possible that this confusion increased because of the three subbands of SB being delivered to the same narrow DB. This could have made /s/ and /ʃ/ sound more similar after the application of Fcomp.

Changes in the pattern of confusions have been reported for many studies of FL, improvements in the identification of some sounds being offset by worsening in the identification of other sounds (Alexander, 2016; Ellis & Munro, 2015b; Kokx-Ryan et al., 2015; Posen, Reed, & Braida, 1993; Robinson et al., 2007, 2009). It is possible that changes in the pattern of confusions have an impact on the effort required in everyday life listening situations, even when the overall identification score remains roughly constant. For example, it is not known how specific changes in the pattern of confusions affect subjective preference and performance in environments where visual (lip reading) and contextual information are available. However, the results of the SSQ-C (discussed later) suggest that any such effects of Fcomp in everyday life were small.

The outcomes of SINFA suggest that, overall, the transmission of acoustic features did not change across conditions. This is consistent with the findings of Robinson et al. (2007) for the FT hearing aid evaluated by them. At the individual level, there were some differences in the transmission of acoustic features in both our data and those of Robinson et al. (2007). Simpson et al. (2006) did not report individual data, but they reported differences in the amount of transmitted information across groups of participants classified according to whether they obtained benefit from FC or not. Effects of the FL processing may be obscured by averaging across participants. Consistent with the consonant-confusion matrices, for most of our participants for whom the transmission of information varied across conditions, improvements in the transmission of one feature were offset by worsening in the transmission of another feature.

Fcomp did improve the detection of word-final /s, z/. This ability is based on sound detection rather than discrimination. Thus, this outcome is consistent with the improvement in estimated audibility achieved with Fcomp for most of the participants. Inspection of the data (see Figure 8) suggests that those participants who did not seem to obtain benefit from Fcomp for this task were those for whom the estimated audibility of the frequency-lowered signals was low or zero, except for P04. Word-final detection of /s, z/ has been shown to improve with other FL schemes, such as FC (Glista et al., 2009; Wolfe et al., 2010, 2011) and FT (Robinson et al., 2007). Conversely, a few studies (John et al., 2014; Wolfe et al., 2015) showed no benefit of FC for final /s, z/ detection. The participants in the studies that did not show a benefit had mild high-frequency hearing loss and thus probably had reasonable audibility of the cues used for detection of word-final /s, z/ even without FL.

The intelligibility of speech in background babble was assessed for six of the participants. Increasing the audible frequency range of speech improves sentence intelligibility for frequency ranges up to at least 7.5 kHz (Baer et al., 2002; Moore, 2016), especially for speech in babble noise that is spatially separated from the target voice (Levy, Freed, Nilsson, Moore, & Puria, 2015; Moore, Füllgrabe, & Stone, 2010). Thus, we expected that the improved audibility provided by Fcomp might improve performance, at least for the spatially separated configuration. However, there was no effect of Fcomp for either spatial configuration. Moore et al. (2010) recorded their stimuli via a KEMAR dummy head (Burkhard & Sachs, 1975) and presented them to participants via headphones, thereby preserving pinna cues. We used hearing aids with microphones located above the pinna. Therefore, no pinna cues were available. The hearing aids had an algorithm that was intended partly to reproduce pinna cues (Launer, Zakis, & Moore, 2016). However, this may not have been enough to preserve the benefit of increasing the audible bandwidth (Levy et al., 2015). The FL itself and the recoding of a large frequency range in the SB to a smaller range in the DB may also have limited the benefit of increasing the audible bandwidth. Finally, it could be that the speech material used here was not sensitive enough to allow detection of differences across conditions. The adaptive sentence list sentences contain contextual information, which makes them easier to identify. The use of lower context sentences, such as the IEEE corpus (Rothauser et al., 1969), might have led to different outcomes.

At the time of performing this study, there were no published studies evaluating Fcomp. Recently, a version of Fcomp implemented in Oticon hearing aids (Angelo, Alexander, Christiansen, Simonsen, & Jespergaard, 2015) was evaluated for a group of adults and children (Kirby et al., 2017). No benefit of Fcomp-on was found for speech intelligibility (monosyllabic words and sentences, VCV test in background noise), plural detection, or listening effort, although the participants preferred the sound quality with Fcomp-on, at least in some situations. Several factors could account for why they found no benefit for plural detection while we found a benefit for /s, z/ detection. These include the use of different hearing aids, different methods for prescribing gain, different methods for scoring plural detection (percent correct as opposed to *d*′ for /s, z/ detection), and the degree of hearing loss of the participants. This last factor is probably the most important. Our participants had more severe hearing loss, and their values of *Thr_f_* (mean of 2.65 kHz for the ears with better audibility) were much lower than the values of the “maximum audible frequency” reported by Kirby et al. (2017). Their values of the maximum audible frequency were close to 4 kHz for the adults and 5.5 kHz for the children. Thus, the audibility of high-frequency information in the control condition was higher for the group tested by Kirby et al. (2017), and a benefit from Fcomp was less likely to occur.

Several previous studies have evaluated FL hearing aids using different types and locations of the background sound. The results are conflicting, some showing benefit of FL (Bohnert et al., 2010; Ellis & Munro, 2015b; Wolfe et al., 2011), some showing no significant effect (Gifford et al., 2007; Hopkins et al., 2014; John et al., 2014; Kokx-Ryan et al., 2015; Picou et al., 2015; Robinson et al., 2009; Simpson et al., 2006; Wolfe et al., 2010, 2015), and some showing deleterious effects (Hillock-Dunn et al., 2014; Miller et al., 2016; Perreau, Bentler, & Tyler, 2013). Differences in FL algorithms, settings of the FL, hearing loss of the participants, type of background noise, and experimental design are likely to underlie the differences across studies.

We obtained a measure of subjective benefit using the SSQ-C questionnaire. We hoped that if any consistent patterns emerged from the outcomes of the SSQ-C, they could be used to guide the selection of outcome measures in future studies. The group average results did not show a preference for Fcomp-on versus Fcomp-off. However, some participants reported that the sound quality of music was adversely affected by Fcomp. The sound quality of music has been shown to be affected by the use of some FL algorithms, such as FC (Mussoi & Bentler, 2015), especially for musically trained listeners and moderate or strong settings of the FC. Despite the inharmonicity produced by FC, there appears to be a range of FC settings that leads to acceptable sound quality for music for hearing-impaired participants (Mussoi & Bentler, 2015; Parsa, Scollie, Glista, & Seelisch, 2013). It has been reported recently that an algorithm combining FC and FT improved the perception of spectral detail in music as well as preserving sound quality (Kirchberger & Russo, 2016). Fcomp does not preserve the harmonic structure of sounds, but despite this most participants did not rate music as having a lower quality with it. In any case, as noted earlier, FL is usually switched off in the music program that is provided with many hearing aids.

Individual variability in the effect of Fcomp was observed for all of the speech tests used in this study. Such variability has been reported for most studies of FL (Bohnert et al., 2010; Ellis & Munro, 2015b; Gifford et al., 2007; Glista et al., 2009; McDermott, Dorkos, Dean, & Ching, 1999; McDermott & Dowell, 2005; Parent, Chmiel, & Jerger, 1997; Picou et al., 2015; Robinson et al., 2007, 2009; Simpson et al., 2006; Simpson, McDermott, & Dowell, 2005; Souza et al., 2013; Turner & Hurtig, 1999). This variability may be related to individual differences in the cues used for speech identification and in the ability to use the new cues provided by FL. Relationships have been reported between amount of high-frequency hearing loss and benefit from FC in a plural detection task (Glista et al., 2009) and between amount of high-frequency hearing loss and benefit from FC in noise (Ellis & Munro, 2015a).

Our participants were aged 66 years or more. The prevalence of cognitive impairment increases with increasing age. One could argue that, if a high proportion of the participants had cognitive impairment, this might have decreased the likelihood of measuring a benefit of Fcomp. However, cognitive ability has been found not to predict the benefit of FC for normal-hearing listeners (Ellis & Munro, 2013) or hearing-impaired listeners (Ellis & Munro, 2015a). It is also possible that older adults are less able to learn to use novel acoustic cues than younger adults or children (Glista et al., 2009).

The presence of DRs was found previously not to be correlated with performance in a sentence-in-noise intelligibility task with and without FC (Ellis & Munro, 2015a). However, for all but two participants, the DRs of the participants tested by Ellis and Munro had relatively high *f_e_* values of 3 or 4 kHz. The impact of DRs on hearing-aid benefit is expected to be low when *f_e_* is relatively high (Moore, 2002), as listeners with DRs can benefit from amplification of frequencies up to nearly an octave above *f_e_*. Thus, the participants of Ellis and Munro would have been able to use information from frequency components over the total bandwidth of the hearing aids used. It is difficult to evaluate the impact of DRs on the performance of our participants, as most of the participants had DRs, and the two with no DRs had much better hearing thresholds than the rest. A study assessing the impact of DRs on the benefit of Fcomp would require an experimental design different from the one used here.

### Limitations of the Current Study

There are some limitations of the present study. One is that the number of participants was small, especially for the speech-in-noise test, for which only six participants could be tested. Another limitation is that although most participants had very similar durations of experience with the two conditions, there were a few who had a slightly longer duration with one than with the other, mainly due to scheduling difficulties. In addition, the total duration of the two test periods varied across participants, which may have contributed to the variability of outcomes across participants. However, Hopkins et al. (2014) showed that, for adult participants, the duration of experience with FC was not correlated with benefit, as assessed using VCVs in quiet and the intelligibility of sentences in noise. Their participants had durations of experience ranging from 1 to 121 months. Conversely, Ellis and Munro (2015b) found that acclimatization to FC did occur for VCVs in noise. Our stimuli were similar to those of Hopkins et al. (2014), so it is likely that the effects of the duration of experience were small. Wolfe et al. (2011) and Glista et al. (2012) reported evidence of acclimatization to FC for children. However, there was no control group in these studies, so it is possible that the changes observed were simply due to maturation and general learning, especially for the children tested by Wolfe et al. (2011), who were aged between 5 and 11 years. Glista et al. (2012) found that acclimatization occurred for some children only. Acclimatization could be different for children and adults because it depends partly on previous experience.

### Concluding Remarks

Fcomp improved the audibility of high-frequency sounds for most participants. The pattern of consonant confusions changed with Fcomp, with improvements being offset by new confusions. Future research should address the fine tuning of the Fcomp algorithm to reduce the number of new confusions while keeping the improvements. Fcomp led to a modest but significant improvement in the detection of word-final /s, z/. The intelligibility of speech in noise was not affected by Fcomp, and ratings of everyday experience did not differ between Fcomp-on and Fcomp-off. Although the evidence for benefits of Fcomp was restricted to the detection of word-final /s, z/, Fcomp did not lead to worse performance for any of the outcome measures. Fcomp, in common with other FL methods, may be useful for clinicians when reductions in high-frequency gain are needed to avoid acoustic feedback. Feedback problems are likely to be reduced both because lower gains are needed with FL stimuli and because frequency shifting is well known as a method of reducing feedback. In addition, the lower output levels that are required when FL is used may help avoid hearing damage and distortion (Ching, Johnson, Seeto, & Macrae, 2013).
